# Autologous formalin-fixed tumor vaccine suppressed re-recurrence of HCV-related hepatocellular carcinoma following 29 unsuccessful treatments with extensive conventional therapy: a case report

**DOI:** 10.1186/1477-7819-10-144

**Published:** 2012-07-12

**Authors:** Toshio Inui, Tadao Ohno

**Affiliations:** 1 Inui Cancer Immunotherapy Clinic, 6-14-17 Kinndachou, Moriguchi-shi, Osaka, 570-0011, Japan; 2 Cell-Medicine, Inc., Sengen 2-1-6, Tsukuba Science City, Ibaraki, 305-0047, Japan; 3 Advanced Research Institute for Science and Engineering, Waseda University, 3-4-1 Okubo, Shinjuku-ku, Tokyo, 169-8555, Japan

**Keywords:** Tumor vaccine, hepatocellular carcinoma, suppression of re-recurrence

## Abstract

Treatment of hepatocellular carcinoma (HCC) with autologous formalin-fixed tumor vaccine after primary resection has been shown to suppress the recurrence of hepatitis B virus-associated HCC, but the effect of this treatment on hepatitis C virus (HCV)-related disease has not yet been clarified. Here, we report a case of a patient with repeat recurrent HCC that was associated with HCV who had endured 29 episodes of HCC recurrence despite a variety of therapy using conventional methods. Finally, treatment with a single course of autologous formalin-fixed tumor vaccine resulted in suppression of potential further re-recurrence of HCC for more than 43 months without any additional treatment.

## Background

Recurrence of hepatocellular carcinoma (HCC) is highly frequent despite curative resection. In a randomized clinical trial, treatment with autologous formalin-fixed tumor vaccine (AFTV) after resection of the monocentric primary HCC reduced the risk of recurrence by 81% (*P* = 0.01) and prolonged median overall survival time (*P* = 0.01) in patients with hepatitis B virus (HBV)-associated HCC [1]. However, we had no clear clinical data on the effect of AFTV on hepatitis C virus (HCV)-related HCC. Here, we report a case of a patient with repeat recurrent HCC that was associated with hepatitis C virus (HCV) who had been unsuccessfully treated 29 times with a variety of conventional methods and endured frequent episodes of recurrent disease. Finally, treatment with a single course of AFTV injection resulted in suppression of potential re-recurrence of HCC for more than 43 months.

## Case presentation

**Figure 1:**
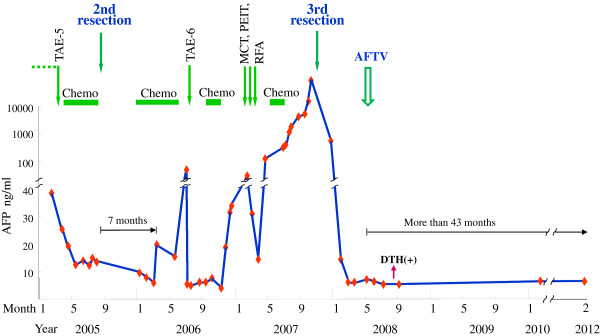
**Alpha-fetoprotein (AFP) levels after the 11th treatment (TAE).** Autologous formalin-fixed tumor vaccine (AFTV) treatment, which consisted of a single course of vaccine injection, was initiated in July 2008 with no further treatment. ‘Chemo’ in the figure indicates chemotherapy with 5-fluorouracil (5FU) and IFNa (the so-called FAIT protocol) [2-4], which was administered as treatments number 12 to 15, 17 to 19, 21, and 27 as shown in Table 1.

**Figure 2:**
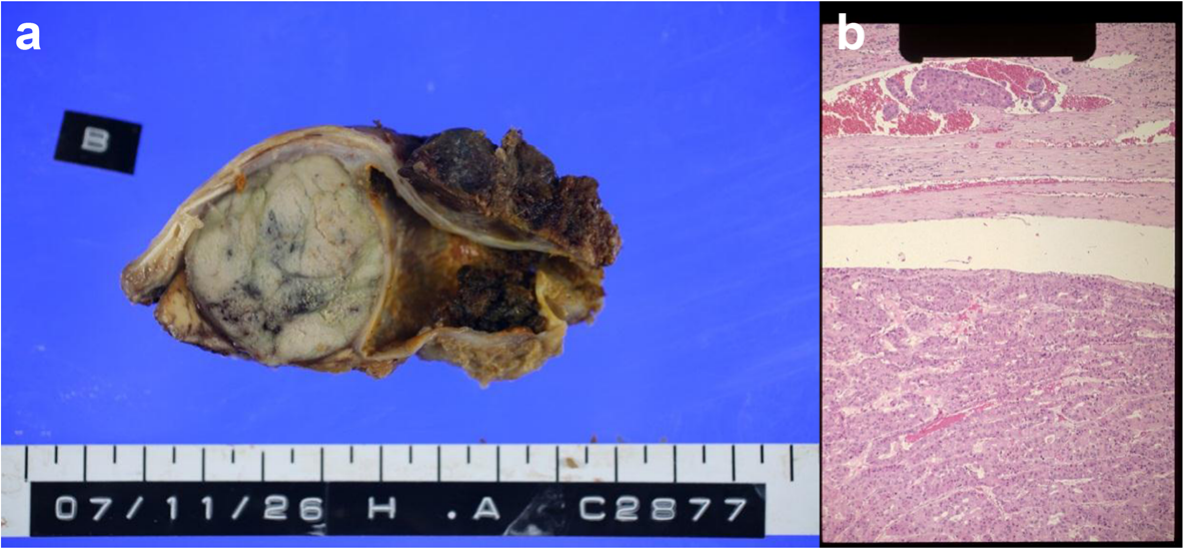
**Re-recurrent tumor in S7 removed during the third resection.** (**a**) The resected 3 cm re-recurrent carcinoma with partial thoracic diaphragm. (**b**) H & E-stained section of the 3 cm re-recurrent carcinoma. Portal vein thrombi were observed.

A seventy-year-old man visited our clinic on June 10, 2008, and presented us with his twenty-nine-treatment history of HCC over a seven-year period as shown in Table 1, although the precise doses of chemotherapeutics were not recorded. Before the first treatment, he was hospitalized in 1964 with acute hepatitis and was subsequently found to be an anti-HCV antibody carrier in 1993. Since then, he had been treated with interferon-alpha (IFNa) and monoammonium glycyrrhizinate. However, in October 1993, it was found that he had developed hepatocellular carcinoma (HCC) in S8 of the liver. As therapy for the HCC, multiple conventional treatments had been applied, such as transarterial embolization (TAE), acetic acid injection, radiofrequency ablation (RFA), surgical resection, transcatheter hepatic artery infusion chemotherapy (TAI) with epirubicin, percutaneous ethanol injection therapy (PEIT), and microwave tumor coagulation (MTC). The first surgery was carried out in November 2004 to remove the tumor, but resulted in incomplete resection. During the second surgery in September 2005, 350 g of the left lobe of the liver was removed to resect a portal vein tumor (Vp4). Further chemotherapy with the so-called FAIT protocol (5-fluorouracil (5FU) plus IFNa) [2-4] was also performed in a clinical trial, and peripheral blood alpha-fetoprotein (AFP) level decreased from 14.2 to 6 ng/ml for six months. However, seven months after the second surgery, the CT image showed recurrent tumors in S7, and the AFP level increased rapidly to 20 ng/ml. Conventional treatments (Table 1) were ineffective and were followed by repeated regrowths of HCC in S7. Further, AFP level increased from 1,143 to 41,958 ng/ml between July 13 and November 21, 2007 (Figure 1). The third aggressive operation was performed together with thoracotomy and partial thoracic diaphragm removal in November 26, 2007, and the tumor was successfully resected (Figure 2a). Pathological examination revealed portal vein tumor thrombi (Figure 2b). The AFP level rapidly decreased to 7 ng/ml by March 6, 2008, where it maintained level for a month, but slightly increased to 8 ng/ml by May 2, 2008.

**Table 1:** Treatments for recurrent tumors of the present case before the AFTV injection

**Treatment number**	**Year. Month**	**Treatments**
1	2001.09	TAE to the 5 cm primary tumor in S8
2	2002.02-03	TAE to recurrent tumor
3, 4, 5	2002.11-12	Acetic acid injection, three times; no effect
6, 7	2003.09	TAE + RFA; tumor shrinkage
8	2004.08	TAE, no effect
9	2004.11	First surgical resection; incomplete resection
10	2004.12	TAI with epirubicin
11	2005.02	TAE for portal vein tumor thrombosis (Vp4)
12, 13, 14, 15	2005.03-07	Chemotherapy with 5FU and IFNa, preliminary course + three courses; no change
16	2005.09	Second surgical resection of portal vein tumor (Vp4)
17, 18, 19	2006.01-05	Chemotherapy with 5FU and IFNa, three courses
20	2006.06	TAE in S7
21	2006.10-11	Chemotherapy with 5FU and IFNa, an additional course
22, 23, 24	2007.02	MTC, PEIT, lipiodol embolization to the recurrent HCC in S7
25, 26	2007.03	RFA + PEIT; no effect
27	2007.05	Chemotherapy with 5FU and IFNa, an additional course
28	2007.08	TAE for 1.2 cm tumor in S7; no effect
29	2007.11	Third surgical resection after thoracotomy and partial thoracic diaphragm removal

5FU, 5-fluorouracil; IFNa, interferon-alpha; HCC, hepatocellular carcinoma; MTC, microwave tumor coagulation; PEIT, percutaneous ethanol injection therapy; RFA, radiofrequency ablation; TAE, transarterial embolization; TAI, transcatheter hepatic artery infusion chemotherapy.

The above treatments were carried out at three different institutions prior to presentation at our clinic and were recorded by the patient himself without precise descriptions. After the 29th treatment, a single course of AFTV injection [5] was initiated in July 2008 with no further treatment.

Thereafter, the patient strenuously insisted to be treated with autologous formalin-fixed tumor vaccine (AFTV) at our clinic. AFTV was prepared as previously reported [5] with four paraffin-embedded HCC pieces derived from the third resection. A single course of AFTV therapy consisted of (i) first, a delayed-type hypersensitivity (DTH) test with the fixed HCC fragments but no immunoadjuvant, which was initiated 48 hours before the first AFTV injection, (ii) intradermal injection of AFTV, two weeks apart and up to three times, and (iii) a second DTH test performed two weeks after the last AFTV injection according to the schedule described [1]. The DTH test was negative before the first AFTV injection and became positive (11 × 12.5 mm erythema and 5 × 5 mm induration) after the third AFTV injection as evaluated on August 25, 2008. We found no adverse effects except for small local erythema at the AFTV injection sites. We also observed his blood HCV-RNA level, 5.7 log IU/mL.

Without any further adjuvant therapy, the patient maintained low AFP levels (6 to 8 ng/ml) for more than 43 months (Figure 1). The patient remains well with no evidence of metastasis or local recurrence.

Complete suppression of HCC recurrence is known to be difficult to attain with conventional treatment methods. In the present case, eight different therapies (Table 1) had been applied with no success, since repeated recurrence of the HCC in S7 of the liver occurred before the AFTV therapy. Although the third surgical operation of the recurrent HCC rapidly decreased AFP levels from 41,958 to 7 ng/ml, it was highly likely that the potentially remaining HCC cells were not completely removed, since multiple portal vein tumor thrombi have been observed (Figure 2b). Also, HCC recurrence rates in patients of positive HCV-RNA were known very high, such as 55.3% at the third year and 72.2% at the fifth year after surgical resection [6]. In this case, recurrent tumors in S7 were found seven months after the second operation, which had decreased the AFP level down to 6 ng/ml. Therefore, we consider that, after the third operation, AFTV treatment successfully suppressed potential further HCC recurrence in this patient.

## Conclusions

The findings from the present case suggest that AFTV treatment suppressed potential further HCC recurrence for more than 43 months after the third resection and that AFTV is a feasible therapy for further large-scale clinical trials for suppression of HCC recurrence that is associated with not only HBV but also HCV after surgical resection.

## Consent

Written informed consent in English and Japanese has been obtained from the patient for publication of this case report and any accompanying images.

## Abbreviations

AFP, alpha-fetoprotein; AFTV, autologous formalin-fixed tumor vaccine; DTH, delayed-type hypersensitivity; 5FU, 5-fluorouracil; IFNa, interferon-alpha; H & E, hematoxylin and eosin; HBV, hepatitis type B virus; HCC, hepatocellular carcinoma; HCV, hepatitis type C virus; MTC, microwave tumor coagulation; PEIT, percutaneous ethanol injection therapy; RFA, radiofrequency ablation; TAE, transarterial embolization; TAI, transcatheter hepatic artery infusion chemotherapy.

## Competing interests

The authors declare that they have no competing interests.

## Authors’ contributions

TO suggested to treat the patient with AFTV, produced the vaccine, and wrote the report. TI carried out the AFTV therapy. All authors read and approved the final manuscript.

## Authors’ information

TI is the owner of an independent clinic in Osaka where he is treating cancer patients mainly with immunotherapy. TO is the president and CEO of Cell-Medicine, Inc., a company for research and development of immunotherapy, and a visiting professor of Waseda University.

## References

[B1] Kuang M Peng BG Lu MD Liang LJ Huang JF He Q Hua YP Totsuka S Liu SQ Leong KW Ohno T Phase II randomized trial of autologous formalin-fixed tumor vaccine for postsurgical recurrence of hepatocellular carcinoma Clin Cancer Res 2004 10 1574 1579 10.1158/1078-0432.CCR-03-0071 15014006

[B2] Nagano H Monden M FAIT (FU arterial infusion and interferon therapy) for hepatocellular carcinoma Nihon Rinsho 2006 64 1314 1318 16838650

[B3] Nagano H Miyamoto A Wada H Ota H Marubashi S Takeda Y Dono K Umeshita K Sakon M Monden M Interferon-alpha and 5-fluorouracil combination therapy after palliative hepatic resection in patients with advanced hepatocellular carcinoma, portal venous tumor thrombus in the major trunk, and multiple nodules Cancer 2007 110 2493 2501 10.1002/cncr.23033 17941012

[B4] Wada H Nagano H Noda T Damdinsuren B Marubashi S Miyamoto A Takeda Y Umeshita K Dono K Sakon M Wakasa K Monden M Complete remission of hepatocellular carcinoma with portal vein tumor thrombus and lymph node metastases by arterial infusion of 5-fluorouracil and interferon-alpha combination therapy following hepatic resection J Gastroenterol 2007 42 501 506 10.1007/s00535-007-2028-x 17671767

[B5] Ishikawa E Tsuboi K Yamamoto T Muroi A Enomoto T Takano S Matsumura A Ohno T A clinical trial of autologous formalin-fixed tumor vaccine for glioblastoma multiforme patients Cancer Sci 2007 98 1226 1233 10.1111/j.1349-7006.2007.00518.x 17517052 PMC11158799

[B6] Ikeda K Kobayashi M Saitoh S Someya T Hosaka T Akuta N Suzuki F Tsubota A Suzuki Y Arase Y Kumada H Recurrence rate and prognosis of patients with hepatocellular carcinoma that developed after elimination of hepatitis C virus RNA by interferon therapy. A closed cohort study including matched control patients Oncology 2003 65 204 210 10.1159/000074472 14657593

